# Consensus recommendations for the diagnosis, treatment and follow-up of inherited methylation disorders

**DOI:** 10.1007/s10545-016-9972-7

**Published:** 2016-09-26

**Authors:** Ivo Barić, Christian Staufner, Persephone Augoustides-Savvopoulou, Yin-Hsiu Chien, Dries Dobbelaere, Sarah C. Grünert, Thomas Opladen, Danijela Petković Ramadža, Bojana Rakić, Anna Wedell, Henk J. Blom

**Affiliations:** 1 Department of Pediatrics, University Hospital Center Zagreb, Kišpatićeva 12, Rebro, 10000 Zagreb, Croatia; 2 University of Zagreb, School of Medicine, Zagreb, Croatia; 3 Department of General Pediatrics, Division of Metabolic Medicine and Neuropediatrics, University Hospital Heidelberg, 69120 Heidelberg, Germany; 4 1st Pediatric Department, Aristotle University of Thessaloniki, Thessaloniki, Greece; 5 Department of Medical Genetics and Pediatrics, National Taiwan University Hospital, Taipei, Taiwan; 6 Medical Reference Center for Inherited Metabolic Diseases, Jeanne de Flandre University Hospital and RADEME Research Team for Rare Metabolic and Developmental Diseases, EA 7364 CHRU Lille, 59037 Lille, France; 7 University Medical Centre Freiburg, Freiburg, Germany; 8 Biochemical Genetics Laboratory, BC Children’s Hospital, 4500 Oak Street, Vancouver, BC V6H 3N1 Canada; 9 Department of Molecular Medicine and Surgery, Karolinska Institutet, Stockholm, Sweden; 10 Centre for Inherited Metabolic Diseases, Karolinska University Hospital, Stockholm, Sweden; 11 Laboratory of Clinical Biochemistry and Metabolism, Department of General Pediatrics Adolescent Medicine and Neonatology, University Medical Centre Freiburg, Freiburg, Germany

## Abstract

Inherited methylation disorders are a group of rarely reported, probably largely underdiagnosed disorders affecting transmethylation processes in the metabolic pathway between methionine and homocysteine. These are methionine adenosyltransferase I/III, glycine N-methyltransferase, S-adenosylhomocysteine hydrolase and adenosine kinase deficiencies. This paper provides the first consensus recommendations for the diagnosis and management of methylation disorders. Following search of the literature and evaluation according to the SIGN-methodology of all reported patients with methylation defects, graded recommendations are provided in a structured way comprising diagnosis (clinical presentation, biochemical abnormalities, differential diagnosis, newborn screening, prenatal diagnosis), therapy and follow-up. Methylation disorders predominantly affect the liver, central nervous system and muscles, but clinical presentation can vary considerably between and within disorders. Although isolated hypermethioninemia is the biochemical hallmark of this group of disorders, it is not always present, especially in early infancy. Plasma S-adenosylmethionine and S-adenosylhomocysteine are key metabolites for the biochemical clarification of isolated hypermethioninemia. Mild hyperhomocysteinemia can be present in all methylation disorders. Methylation disorders do not qualify as primary targets of newborn screening. A low-methionine diet can be beneficial in patients with methionine adenosyltransferase I/III deficiency if plasma methionine concentrations exceed 800 μmol/L. There is some evidence that this diet may also be beneficial in patients with S-adenosylhomocysteine hydrolase and adenosine kinase deficiencies. S-adenosylmethionine supplementation may be useful in patients with methionine adenosyltransferase I/III deficiency. Recommendations given in this article are based on general principles and in practice should be adjusted individually according to patient’s age, severity of the disease, clinical and laboratory findings.

**Electronic supplementary material:**

The online version of this article (doi:10.1007/s10545-016-9972-7) contains supplementary material, which is available to authorized users.

## Introduction

**Fig. 1:**
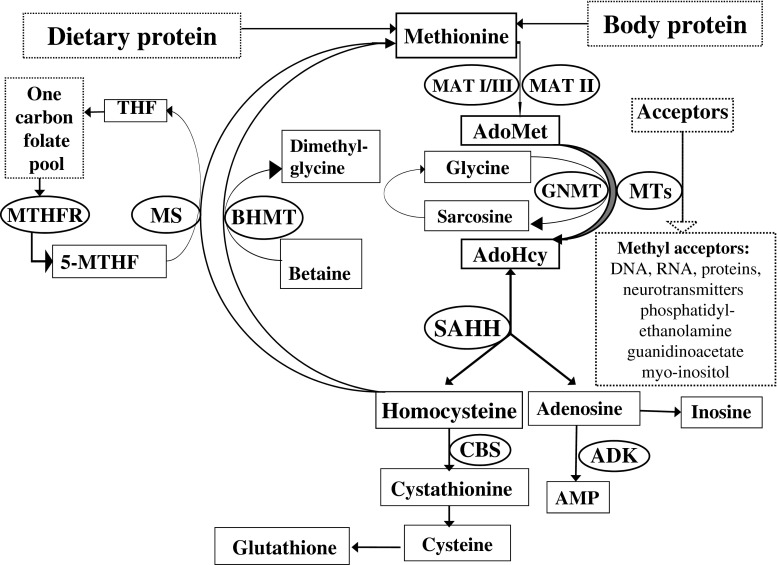
Methionine metabolism. AdoMet, *S*-adenosylmethionine; AdoHcy, *S*-adenosylhomocysteine; THF, tetrahydrofolate; 5-MTHF, 5-methyltetrahydrofolate; AMP, adenosine monophosphate. The following enzymes are in circles: MAT, methionine adenosyltransferase (E.C.2.5.1.6); GNMT, glycine *N*-methyltransferase (E.C.2.1.1.20); MTs, a variety of AdoMet-dependent methyltransferases; SAHH, AdoHcy hydrolase (E.C.3.3.1.1); CBS, cystathionine β-synthase (E.C.4.2.1.22); MS, methionine synthase (5-MTHF-homocysteine methyltransferase) (E.C.2.1.1.13); BHMT, betaine-homocysteine methyltransferase (E.C.2.1.1.5); MTHFR, methylenetetrahydrofolate reductase (E.C.1.5.1.20); ADK, adenosine kinase (E.C.2.7.1.20)

Inherited methylation disorders are a group of disorders affecting the transmethylation processes in the metabolic pathway between methionine and homocysteine (Fig. 1). Transmethylation is the transfer of a methyl group from methionine via S-adenosylmethionine (AdoMet), which is the methyl group donor in humans, to numerous acceptors. The large group of methyl group acceptors includes important molecules such as DNA, RNA, lipids, proteins and amino acids, neurotransmitters and guanidinoacetate (Clarke and Banfield 2001). Methyl group transfer is mediated by numerous different methyltransferases. Katz and co-workers (Katz et al 2003) concluded from bioinformatic analysis of various genomes, including that of humans, that class-1 AdoMet-dependent methyltransferases correspond to about 0.6 to 1.6 % of all open reading frames in analysed genomes. This would point to about 300 AdoMet-dependent methyltransferases in humans. The fact that transmethylation, together with the process of remethylation of homocysteine to methionine, i.e. the methionine cycle, occurs in almost all mammalian tissues and cells, also reflects its significance for normal cellular hemostasis (Mudd et al 2001a; Finkelstein 2006; Brosnan and Brosnan 2006; Blom et al 2006).

Methylation reactions are influenced by changes in the concentrations of AdoMet and S-adenosylhomocysteine (AdoHcy), which may be reflected by their ratio (the so-called methylation index). Since these are directly affected in inherited methylation disorders, it is easily understandable that the pathogenetic mechanisms of these disorders are complex, still poorly understood and that many organs and body functions may be affected. Therefore, inherited methylation disorders should be included in the differential diagnosis of many signs and symptoms, particularly concerning neurological, muscle and liver function. A main aim of the “European network and registry for homocystinurias and methylation defects (E-HOD)” project is to provide recommendations for diagnosis and treatment for this rare group of disorders. Since most physicians will not have any experience with methylation disorders, they are probably largely underdiagnosed. In addition there is a lack of validated and uniform therapeutic strategies and follow-up approaches.

This paper deals with four inherited enzymopathies. Three of these directly affect the reactions in the methionine pathway. The first is the conversion of methionine to AdoMet, catalyzed by methionine adenosyltransferase (MAT; E.C. 2.5.1.6). MAT has three isoforms. MAT I and MAT III are liver specific, while MAT II is present mainly in fetal liver and extrahepatic tissues. Both MAT I (a tetramer) and MAT III (a dimer) are coded by the *MAT1A* gene, while MAT II is coded by the *MAT2A* gene. Human disease can be caused by mutations in the *MAT1A* gene, resulting in MAT I/III deficiency (also called Mudd’s disease).^1^ To our knowledge, MAT II deficiency has not been described as a human disease. Individuals with a mutation in one of their *MAT2A* alleles may be predisposed to thoracic aortic aneurysms (Guo et al 2015). The second enzyme is glycine N-methyltransferase (GNMT; E.C. 2.1.1.20) which transfers a methyl group to glycine producing sarcosine. GNMT deficiency causes elevation of AdoMet, as demonstrated both in humans and knockout mice (Mudd et al 2001b; Augoustides-Savvopoulou et al 2003; Rakic et al 2015; Barić et al 2016; Luka et al 2006). This is the only methyltransferase deficiency which is known to cause elevation of AdoMet, and therefore the only methyltransferase deficiency included in this article. GNMT is abundantly expressed in the liver and regulates the degradation of excess methyl groups, and is therefore a main regulator of methionine metabolism (Yeo and Wagner 1994). The third enzyme, S-adenosylhomocysteine hydrolase (SAHH; E.C. 3.3.1.1), is a homotetrameric enzyme, which converts S-adenosylhomocysteine to homocysteine and adenosine. Adenosine kinase (ADK; E.C.2.7.1.20) is not a direct part of the transmethylation process. It catalyzes the phosphorylation of adenosine to adenosine monophosphate and its deficiency causes the accumulation of adenosine, which subsequently leads to elevation of AdoHcy and AdoMet thus disrupting the methionine cycle and affecting the methylation processes. Therefore, ADK deficiency results in a disorder of methylation and is thus included in this guideline.

## Methods

Producing recommendations for diagnosis and treatment of the methylation disorders was one of the aims of the E-HOD project. To assure optimal expertise, experts within the E-HOD network and authors of publications related to patients with inherited methylation disorders have been invited to participate in this work. Standard systematic literature searches and evaluations of the quality of evidence for guidelines/recommendations production were performed according to the SIGN methodology (for details see Supplementary material). Due to the extreme rarity of most disorders we had to simplify this approach. Thorough evaluation of all published patients was complemented by the analysis of individual and sporadic unpublished data (see Supplementary material for more details).

Methylation disorders have been very rarely reported, either as case reports or case series. We are aware of a total of five patients with GNMT deficiency, four reported *in extenso* in three publications (Mudd et al 2001b; Augoustides-Savvopoulou et al 2003; Barić et al 2016), nine patients with SAHH deficiency reported in seven publications in detail (Barić et al 2004, 2005; Buist et al 2006; Grubbs et al 2010; Honzík et al 2012; Strauss et al 2015; Stender et al 2015) and 21 patients with ADK deficiency, 20 of them reported in four articles *in extenso* (Bjursell et al 2011; Labrune et al 1990; Staufner et al 2016a, b). Very recently, almost all known patients with MAT I/III deficiency (in total 64 patients) were extensively described (Chien et al 2015).

## Methionine adenosyltransferase I/III deficiency

### Diagnosis

#### Selective screening

##### Clinical presentation

Patients may present with neurodevelopmental problems and/or malodorous breath but most patients have been detected in newborn screening programs (see also below). In the recent review describing most known patients with MAT I/III deficiency, 58 out of 64 patients were ascertained by high methionine levels in newborn screening for CBS deficiency or via family members, and the remaining were diagnosed via selective screening (Chien et al 2015).

About half of the patients with autosomal recessive *MAT1A* mutations have developed neurological symptoms later in life, so MAT I/III deficiency cannot be considered to be a benign genetic disorder (Chien et al 2015). The reported symptoms, which should accordingly be included in the differential diagnosis, were mostly neurological such as subnormal psychomotor development or intelligence, tremor, dystonia, dysmetria, severe headaches, nystagmus, dysdiadochokinesis, increased tendon reflexes, language difficulties and learning disabilities (Surtees et al 1991; Chamberlin et al 2000; Mudd et al 1995, 2001a). In addition, hypermethioninemia can be associated with an unusual breath odour, presumably due to formation of dimethylsulfide via transamination of methionine (Gahl et al 1988). Neurological abnormalities have occurred particularly in patients with plasma methionine concentrations above 800 μmol/L, whereas they have been rare in subjects with lower levels (Chien et al 2015). Although the majority of patients have normal brain images, some brain imaging findings, in particular when associated with attributable clinical signs and symptoms or hypermethioninemia, should also suggest MAT I/III deficiency. Typical changes are demyelination with oedema of subcortical and deep white matter, more pronounced in the dorsal brain stem and resulting in separation of myelin layers—the so-called vacuolating myelinopathy (Chamberlin et al 1996; Tada et al 2004; Braverman et al 2005). Grey matter and basal ganglia involvement has also been described (Chamberlin et al 2000).

MAT I/III is a liver specific enzyme and liver histology was studied in a few MAT I/III-deficient patients. Light microscopy was usually normal, but electron microscopy showed some abnormalities, including mitochondria of abnormal shape or breaks in their outer membranes and increased smooth and decreased rough endoplasmic reticulum. Increased pericellular collagen fibres were present in the space of Disse. Liver function tests in patients have been normal so far (Chien et al 2015) although development of liver dysfunction in the long term remains a theoretical possibility.

##### Biochemical abnormalities

MAT I/III deficiency is the most common cause of persistent isolated hypermethioninaemia.

*Statement #1. Grade of recommendation C*

MAT I/III deficiency should be suspected in all persons with unexplained hypermethioninemia.

*Statement #2. Grade of recommendation D*

In persons with unexplained neurological signs and symptoms, disorders of methylation should be investigated at least by analysis of plasma amino acids.

#### Differential diagnosis

Hypermethioninemia is often the first finding leading to suspicion of MAT I/III deficiency. Firstly, hypermethioninemia due to CBS deficiency is excluded by measuring total homocysteine (tHcy). If tHcy is normal or only slightly increased, sequencing the *MAT1A* gene is the direct way to diagnosis. MAT I/III is only expressed in liver, making enzymatic diagnostics impractical.

Other defects in methylation can be excluded by determining plasma AdoMet and AdoHcy which are elevated in other methylation disorders (GNMT, SAHH, ADK deficiency) and also in CBS deficiency. Patients with MAT I/III deficiency usually have normal AdoMet and AdoHcy levels in plasma. It may be confusing that AdoMet can be higher in the first months of life, perhaps due to incomplete decline of MAT II expression in the liver after birth. Measurement of AdoMet and AdoHcy is only available in specialized centres.

A number of genetic causes of hypermethioninemia other than those covered in these guidelines include heterozygosity for MAT I/III deficiency and several inherited diseases affecting liver function, including citrin deficiency, galactosemia and tyrosinemia type I. Furthermore several non-genetic conditions such as liver disorders or consumption of high methionine containing infant formula and even prematurity can cause hypermethioninemia. Thus exclusion of these causes is essential in the diagnostic process.

*Statement #3. Grade of recommendation D*

Isolated hypermethioninemia should be followed up by measurement of tHcy in plasma and if this is normal or slightly to moderately elevated, plasma AdoMet and AdoHcy should be measured.

*Statement #4. Grade of recommendation C*

Sequencing the *MAT1A* gene is recommended to confirm the diagnosis of MAT I/III deficiency.

#### Newborn screening

In several countries methionine is used as a marker in newborn screening to detect patients with homocystinuria due to CBS deficiency (Mudd 2011), although the sensitivity and the specificity for detecting CBS deficiency is poor, in particular for B_6_-responsive CBS deficient patients (Huemer et al 2015).

The median dried blood spot concentrations of methionine in 143 MAT I/III-deficient individuals (including carriers) in the “Region 4 Stork (R4S) Collaborative Project” database was 103 μmol/L compared to 20 μmol/L in healthy neonates (McHugh et al 2011). A target cut-off value for methionine to differentiate between the 99th centile in healthy newborns and the 5th centile in confirmed true positive cases is in the range of 39 to 50 μmol/L (Huemer et al 2015). The number of patients with MAT I/III deficiency missed in newborn screening is not known. Taken together, clinical and laboratory data are insufficient to develop recommendations on newborn screening for MAT I/III deficiency (Huemer et al 2015).

Once a newborn with elevation of methionine is detected, the next step in clinical assessment is the determination of plasma tHcy, optimally by a second tier test using the original dried blood spot (Turgeon et al 2010), although only very few labs offer this assay. A common cause of elevated methionine in newborn screening is the autosomal dominant form of hypermethioninemia due to monoallelic mutations of *MAT1A* (Chamberlin et al 1997). The most often reported dominant mutation is c.791G > A (p.R264H) (Chien et al 2005; Martins et al 2012; Couce et al 2013) but other *MAT1A* mutations may also cause dominant hypermethioninemia (Fernandez-Irigoyen et al 2010). Blood methionine measurements in parents can help to identify dominant hypermethioninemia although sometimes this may only be slightly elevated. Strong evidence indicates that heterozygotes, in particular c.791G > A (p.R264H) heterozygotes will be clinically unaffected (Couce et al 2013; Chadwick et al 2014). See further under ‘differential diagnosis’ above. A baby of a mother with severe hypermethioninemia may show transiently elevated methionine levels.

*Statement #5. Grade of recommendation D*

Most patients with MAT I/III deficiency have been detected by newborn screening. Due to potentially harmful consequences of MAT I/III deficiency and relatively effective therapeutic options, neonatal screening for this disease may ultimately be justified. However, more clinical and laboratory data are required to make a firm recommendation to initiate newborn screening.

*Statement #6. Grade of recommendation D*

The most common cause for hypermethioninemia detected by newborn screening is a dominant form of hypermethioninemia due to heterozygosity for mutations in *MAT1A*. This condition is considered benign.

#### Prenatal diagnosis

Prenatal diagnosis can be made using *MAT1A* gene analysis. Enzyme assay in amniocytes or chorionic villi, as well as measurement of metabolites in amniotic fluid, have not been tested. The necessity for prenatal diagnosis should be carefully considered before planning the procedure, since a substantial number of patients with MAT I/III deficiency seem to be asymptomatic, and since in the remaining patients the disease is often treatable.

*Statement #7. Grade of recommendation D*

Prenatal testing in this disease is feasible via *MAT1A* gene analysis. Prior to testing, it is essential that the index case has been confirmed genetically, and that the carrier status of the parents is confirmed by mutation analysis. The necessity for prenatal diagnosis requires thoughtful discussion as a substantial number of patients with MAT I/III deficiency seem to be asymptomatic, and since in the remaining patients the disease is often treatable.

### Therapy

Theoretically, there are two therapeutic options: methionine restriction and oral administration of AdoMet.

As described above, neurological abnormalities have tended to occur in patients with plasma methionine concentrations above 800 μmol/L, whereas they have been rare in subjects with lower levels (Chien et al 2015). In some patients the use of a methionine restricted diet seemed to be clinically beneficial (Chien et al 2015). Therefore, if the mean methionine level is above 800 μmol/L, dietary methionine restriction should be considered, even in asymptomatic individuals, aiming at methionine concentrations around 500 to 600 μmol/L (Chien et al 2015). A more rigid methionine restricted diet is difficult to comply to and it does not seem to be clinically beneficial. Theoretically, lowering methionine concentrations below 500 μmol/L may further limit the flux through MAT I/III, if some residual MAT I/III activity remains, which would further decrease the availability of AdoMet (Mudd 2011).

In all patients, possible development of central nervous system signs and symptoms should be kept in mind and regular neurological and cognitive testing should be performed. If indicated, brain MRI should be done. Symptomatic patients should be treated with a methionine restricted diet if the methionine level is above 600 μmol/L, again aiming at methionine levels around 500 to 600 μmol/L.

AdoMet supplementation may be indicated if clinical signs and symptoms develop while on methionine restriction. The most effective oral dose of AdoMet is unknown. Doses between 400 to 1600 mg/day have been used in various conditions and AdoMet seems to be well tolerated. The available evidence suggests that AdoMet supplementation may lead to normalization of brain imaging changes (Chien et al 2015).

*Statement #8. Grade of recommendation D*

If plasma methionine concentrations are above 800 μmol/L, a methionine restricted diet is recommended, with the aim to maintain methionine levels around 500–600 μmol/L, even in asymptomatic individuals.

Symptomatic patients with methionine levels between 600 and 800 umol/L should aim for methionine levels around 500–600 μmol/L.

If the mean plasma methionine is below 500–600 μmol/L, treatment seems not to be indicated.

*Statement #9. Grade of recommendation D*

Besides methionine restriction AdoMet supplementation may be considered as a treatment option.

*Statement #10. Grade of recommendation C*

If clinical signs or symptoms develop while on methionine restriction, supplementing with AdoMet should be considered.

### Follow-up

#### Monitoring

Methionine should be measured at regular intervals, the first years of life around every 2 to 3 months, and later in life every 6 to 12 months. Exact timing for monitoring of methionine concentrations depends on each patient’s individual values and the risk to have concentrations above 800 μmol/L.

In patients on a methionine restricted diet, regular assessments of protein status, amino acids and AdoMet are indicated.

About half of the patients with autosomal recessive *MAT1A* mutations have developed central nervous system complications (Chien et al 2015). Therefore, neurological and cognitive testing should be performed regularly in all patients. We suggest that neurological testing should take place maybe once every 2 to 3 months in infants, and every 6 to 12 later in life. If indicated, brain MRI should be performed.

*Statement #11. Grade of recommendation C-D*

Methionine should be measured at regular intervals, the first years of life around every 2 to 3 months, and later in life every 6 to 12 months. Regular monitoring of methionine concentrations also depends on each patient’s individual values and their potential for concentrations above 800 μmol/L.

*Statement #12. Grade of recommendation D*

Neurological and cognitive testing should be performed regularly in all patients. We suggest that neurological testing should take place once every 2 to 3 months in infants, and every 6 to 12 months later in life. If indicated, brain MRI should be performed.

#### Complications

Central nervous system complications are described in the section on clinical presentation.

## Glycine N-methyltransferase deficiency

### Diagnosis

#### Selective screening

##### Clinical presentation

GNMT deficiency in humans is an autosomal recessive defect which was discovered 15 years ago (Mudd et al 2001b). However, to date, only five individuals from four families have been diagnosed, four of whom have been described in detail (Mudd et al 2001b; Augoustides-Savvopoulou et al 2003; Barić et al 2016) and one in an abstract (Rakic et al 2015). The fact that three of the individuals 18, 15 and 17 years after diagnosis remain clinically well (personal communications R. Cerone and P. Augoustides-Savvopoulou respectively) favours the view that this is a benign disorder in humans. However, taking into account observations that highly elevated plasma methionine itself is a risk factor for development of neurological signs and symptoms (see MAT I/III deficiency above), and that individuals with GNMT deficiency can have plasma methionine concentrations in the range which has been shown to be associated with clinical problems, signs and symptoms related to high methionine can be anticipated (Braverman et al 2005; Mudd 2011).

Based on the findings in the five diagnosed individuals, characteristics of GNMT deficiency are mild to moderate fluctuating elevations of serum aminotransferases and lack of clinical symptoms—the only clinical sign present in two individuals (siblings) being mild hepatomegaly. Presenting symptoms in three individuals were upper respiratory tract infection, failure to thrive and febrile convulsions, respectively. It is unclear whether these symptoms were related to GNMT deficiency. Of note is that aminotransferase levels within the reference range can occur (as observed in two unrelated individuals) and does not exclude the diagnosis. Liver biopsy was performed in two of the five individuals—one with hepatomegaly had mild centrilobular fibrosis with eosinophilic infiltration and one without hepatomegaly had essentially normal findings, showing only mild hydropic degeneration of a few hepatic cells. Liver ultrasound in this individual showed increased echogenicity of periportal spaces only. In all five individuals the unexplained hyperaminotransferasemia (i.e. not attributable to conventional causes of liver disease) triggered the metabolic workup which led to the correct diagnosis.

##### Biochemical abnormalities

Assays of plasma amino acids revealed isolated marked hypermethioninemia ranging from 400 to 1247 μmol/L (ref. range 13–45). Further investigation revealed remarkably high levels of plasma AdoMet in all five individuals (1149–2840 μmol/L, ref. range 73–109), with plasma AdoHcy levels within the normal range (21–72 μmol/L, ref. range 15–45). Plasma sarcosine (N-methylglycine) was measured in three patients (Mudd et al 2001b; Augoustides-Savvopoulou et al 2003) and was well within the reference range (1.4-2.1 μmol/L, ref. range 0.6-2.7). Hypermethioninemia accompanied by very high levels of plasma AdoMet in the presence of normal plasma AdoHcy and sarcosine are considered diagnostic hallmarks of GNMT deficiency. Interestingly, in one child AdoMet levels in whole blood were in the upper normal range, although with an increased AdoMet/AdoHcy ratio (Barić et al 2016). However, in plasma, AdoMet was highly elevated—3348 nmol/L (reference range 71–118 nmol/L), while AdoHcy was 63 nmol/L (reference range 9.3–14.1 nmol/L). A plausible explanation for increased AdoHcy in this individual could be the extremely high plasma AdoMet which may have caused a secondary increase of AdoHcy via non-enzymatic or artefactual decay of AdoMet. In fact, AdoMet is an unstable compound and can break down to AdoHcy due to inappropriate handling or storage, particularly if the sample is not properly acidified. This also shows that plasma is the sample of choice for assaying AdoMet and AdoHcy. This individual also differed in the fact that mild elevations of plasma tHcy levels (21.4-28.3 μmol/L, ref. range 5–12) were found which can be explained by the inhibitory effect of high methionine levels on betaine-homocysteine-methyltransferase, N5-methyltetrahydrofolate–homocysteine methyltransferase and cystathionine gamma-lyase activity (Barić et al 2016). The diagnosis in all five individuals was confirmed by *GNMT* mutation analysis. Three individuals, including the two siblings, were compound heterozygotes for two missense mutations c.1481 T > C (Leu49Pro), c.3715C > A (His176Asn) and c.529C > A (His177Asn), c.431C > T (Ala144Val), respectively (Luka et al 2002; Rakic et al 2015). The His176Asn and His177Asn mutations are in fact the same variant, albeit reported differently by molecular clinical laboratories. Two individuals were homozygous, one for the c.3415A > G (Asn140Ser) mutation (Augoustides-Savvopoulou et al 2003) and the other for the c.296G > A (Arg99His) mutation (Barić et al 2016). Expression studies in three individuals revealed decreased activity for the mutations as follows: Leu49Pro 10 %, His176Asn 75 %, and Asn140Ser 0.5 % of wild type activity (Luka and Wagner 2003) and in one (Arg99His) minimal activity (Barić et al 2016). Predictions of *in vivo* enzyme activities on the basis of activities found in expression studies can be difficult. Kinetic factors and protein conformational changes as possible causes of impaired enzyme function have been studied for mutations Leu49Pro, His176Asn and Asn140Ser, and were discussed in detail (Luka and Wagner 2003).

Thus the initial approach to diagnosis of GNMT deficiency is clinical suspicion of asymptomatic individuals with permanent or intermittent elevation of aminotransferases (with or without hepatomegaly) followed by measurement of plasma amino acids and tHcy. The presence of marked isolated hypermethioninemia should be followed up by measurement of plasma AdoMet and AdoHcy concentrations (including the AdoMet/AdoHcy ratio) and where possible sarcosine, as normal levels of AdoHcy and sarcosine in the presence of greatly increased AdoMet levels are a strong indicator of GNMT deficiency (Mudd 2011). Diagnosis of GNMT deficiency should be confirmed by *GNMT* mutation analysis and, when necessary, expression studies. Enzyme assay is possible but requires a liver biopsy.

*Statement #13. Grade of recommendation C*

Clinical characteristics of GNMT deficiency are mild to moderate fluctuating elevations of aminotransferases (with or without hepatomegaly) and lack of symptoms. Aminotransferase levels in the reference range can occur and do not exclude the diagnosis.

*Statement #14. Grade of recommendation D*

The cornerstone of diagnosis is awareness of GNMT deficiency and increased clinical suspicion of asymptomatic individuals with unexplained elevations of aminotransferases. The initial laboratory approach is measurement of plasma amino acids and tHcy. The presence of persistent marked isolated hypermethioninemia should be followed by assays of plasma AdoMet and AdoHcy concentrations paying attention to the AdoMet/AdoHcy ratio and if possible plasma sarcosine. The diagnosis should be confirmed by *GNMT* mutation analysis and where necessary, expression studies.

#### Differential diagnosis

**Fig. 2:**
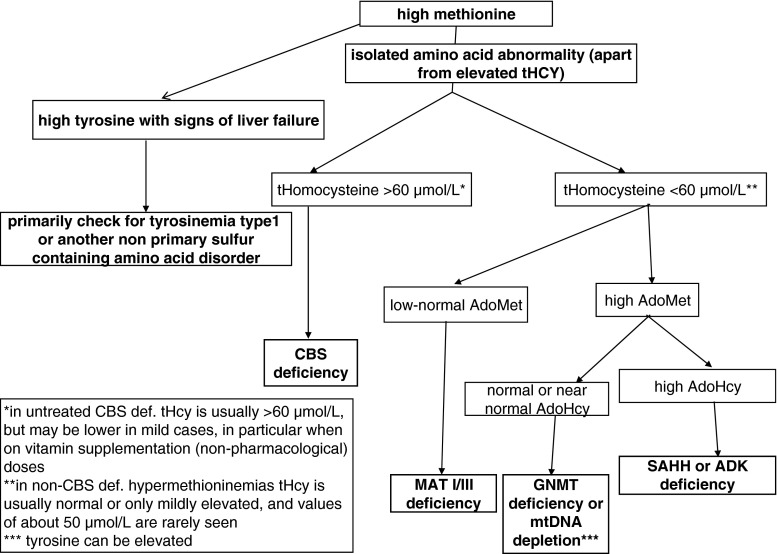
Diagnostic flow-chart in patients with hypermethioninemia (extracted from Barić and Fowler 2014 and slightly modified)

Differential diagnosis should include other causes of hypermethioninemia, genetic and acquired. In contrast to GNMT deficiency most of these disorders are characterized by a range of clinical findings. Careful clinical evaluation combined with key laboratory tests should facilitate their exclusion (see flow chart, Fig. 2).

#### Newborn screening

At the present time, GNMT deficiency does not fulfil the criteria for inclusion in newborn screening programs (Wilson and Jungner 1968; Andermann et al 2008). Furthermore it is unknown whether methionine levels are increased in newborns with GNMT deficiency although the normal blood spot methionine level (19.5 μmol/l, ref. values 15–47) obtained at newborn screening in one patient (Rakic et al 2015) may indicate that hypermethioninemia is not present from birth. AdoMet cannot be used as a newborn screening analyte due to its chemical instability.

*Statement #15. Grade of recommendation D*

Based on the present knowledge, GNMT deficiency does not fulfil the criteria for inclusion in newborn screening programs.

#### Prenatal diagnosis

Prenatal diagnosis of GNMT deficiency is possible (with identification of *GNMT* mutations of the index case) but based on the clinical data of the five diagnosed patients, is irrelevant.

*Statement #16. Grade of recommendation D*

To date, GNMT deficiency in humans is an apparently benign disorder, thus prenatal diagnosis although possible is not recommended.

### Therapy

The necessity for treatment of this asymptomatic seemingly benign disorder is presently questionable, careful follow-up being the main parameter for optimal care. Theoretically, correction of the biochemical abnormality to suppress possible adverse effects would be the therapeutic target, and as evidenced in the first proven individual with GNMT deficiency a low methionine diet (300 mg/day) can correct the biochemical abnormalities (Mudd et al 2001b). However, subjecting asymptomatic individuals to a methionine restricted diet (in infancy 15-20 mg/kg/day) is questionable and has potentially deleterious consequences, e.g. methionine deficiency and related consequences of protein malnutrition and effects on the methionine pathway (Mudd 2011; Barić et al 2016). On the other hand, central nervous system complications (vacuolating myelinopathy) have been described in patients with high levels of methionine (>1000 μmol/L), independent of underlying etiology (Braverman et al 2005; Mudd 2011). To decrease this risk it has been proposed that methionine restriction should be implemented when levels exceed 800 μmol/L (Chien et al 2015), but this cut-off is arbitrary. The aim of the diet should be to maintain methionine levels around 500–600 μmol/L. Recent findings suggest that a potential therapeutic approach may be nicotinamide, which is a substrate for alternative AdoMet dependent methyltransferases. This amide derivative of nicotinic acid has been shown to prevent steatosis and liver fibrosis and to lower the AdoMet levels in *Gnmt* k/o mice. In addition, it prevented DNA hypermethylation and expression of a variety of genes involved in various deleterious processes, e.g. oxidative stress (Varela-Rey et al 2010). However, all of these pathological changes which can be prevented in mice have never been reported in humans with GNMT deficiency.

### Follow-up

#### Monitoring

##### Clinical monitoring

Although GNMT deficiency in humans seems to be a benign disorder this does not appear to be the case in k/o mouse models of GNMT deficiency. The initial findings in these mice confirmed the biochemical abnormalities seen in humans as well as the importance of GNMT for regulation of the AdoMet/AdoHcy ratio and cellular methylation activity (Luka et al 2006, 2009). Recently, elevated aminotransferases, chronic hepatitis (Liu et al 2007), liver steatosis (Liao et al 2012; Zubiete-Franco et al 2015), atherosclerosis (Chen et al 2012), liver fibrosis and most importantly hepatocellular carcinoma (Chen et al 1998; Avila et al 2000; Tseng et al 2003; Martinez-Chantar et al 2008; Varela-Rey et al 2009; Lu and Mato 2012), prostatic cancer (Song et al 2013; Chen et al 2014; Yen et al 2013) and pancreatic ductal adenocarcinoma (Botezatu et al 2015) have been reported and indicate that independent of its metabolic properties, GNMT is also a regulator of cellular proliferation.

Physicians following patients with GNMT deficiency should be aware that besides the liver, GNMT protein is expressed in a number of tissues (prostate, exocrine tissue of the pancreas, proximal kidney tubules, submaxillary glands, intestinal mucosa, cortical neurons and Purkinje cells of the brain) (Luka et al 2009) and that the increasing ongoing research of its functional diversity may be of potential significance for the prognosis and hence monitoring of these patients.

##### Biochemical monitoring

As GNMT deficient patients can have very high methionine levels which have been related to central nervous system complications, monitoring of plasma methionine levels is recommended.

Although none of the five patients with GNMT deficiency show any apparent signs of clinical or biochemical deterioration, in view of the findings in *Gnmt* k/o mouse models (see above), regular measurements of aminotransferases, synthetic liver function (albumin, prothrombin time) and plasma methionine are recommended. It may be advisable to include yearly assessments of plasma alpha-fetoprotein levels as well as liver ultrasound.

*Statement #17. Grade of recommendation D*

Clinicians should be aware of the potential central nervous system complications of severe hypermethioninemia and thoroughly assess the patient, neurologically as well as by plasma methionine monitoring, at intervals depending on age and clinical course.

*Statement #18. Grade of recommendation D*

Due to the limited experience of GNMT deficiency in humans and liver findings in *Gnmt* k/o mice, GNMT deficient individuals should be monitored on a regular basis with measurements of aminotransferases, synthetic liver function, serum alpha-fetoprotein and liver ultrasound in intervals depending on age and clinical course.

#### Complications

To date, complications of GNMT deficiency in humans have not been observed.

## S-adenosylhomocysteine hydrolase deficiency

### Diagnosis

#### Selective screening

The **clinical suspicion** of S-adenosylhomocysteine hydrolase deficiency is guided by findings in ten reported patients from six families (Barić et al 2004, 2005; Buist et al 2006; Ćuk et al 2007; Grubbs et al 2010; Honzík et al 2012; Strauss et al 2015; Stender et al 2015). Two sibs presented severely with fetal hydrops, insufficient liver synthetic function and muscular hypotonia leading to respiratory failure and death in early infancy. Both sisters had cerebellar and pontine hypoplasia, hypoplastic corpus callosum and hypomyelination (Grubbs et al 2010). Muscle disease with increased CK activity was also present in all patients with a relatively milder phenotype. In addition, they had various combinations of developmental delay, behavioural disorders, microcephaly, myelination delay, strabismus, coagulopathy and liver disease. Muscle histology in several patients suggested dystrophic processes.

A novel, very recently reported dimension of the disease is the development of hepatocellular carcinoma, which occurred in a female patient at the age of 29 years and her brother, who likely had SAHH deficiency (Stender et al 2015). SAHH deficiency was diagnosed in the female *post mortem* by whole exome sequencing. Her son, harbouring the same homozygous missense mutation as his mother, was diagnosed with SAHH deficiency at the age of 7 years. He was reported to be asymptomatic, although he had mildly to moderately elevated aminotransferases, decreased albumin and markedly elevated serum levels of methionine, AdoMet and AdoHcy. CK activity was not reported.

*Statement #19. Grade of recommendation C-D*

SAHH deficiency should be suspected in patients with any combination of myopathy with markedly increased CK activity, being the most constant feature, followed by hypotonia, developmental delay, hypomyelination, behavioural problems, liver disease (including hepatocellular carcinoma), coagulation disorders, strabismus and fetal hydrops with brain abnormalities.

*Statement #20. Grade of recommendation C*

SAHH patients may also be asymptomatic.

**Biochemical abnormalities** include elevated aminotransferases and CK activities and variable coagulation abnormalities. Repeatedly reported were: prolonged prothrombin time, low fibrinogen, antithrombin III, protein S, factor II and particularly factor VII, and increased factor V. *Specific abnormalities* pointing to the diagnosis are markedly elevated plasma AdoMet and particularly elevated plasma AdoHcy with normal or near normal tHcy. Plasma methionine can be normal in early infancy, but after that period in untreated patients it is probably constantly elevated. However, since hypermethioninemia is not a constant finding, it is essential to measure AdoMet and AdoHcy in all cases with clinical suspicion of the disorder. In reported patients plasma AdoMet was ∼18-50-fold elevated (up to 5109 nmol/L, reference range 91 ± 28), while AdoHcy was ∼12-200-fold elevated (up to 8139 nmol/L, reference range 27 ± 15). In early infancy these elevations can be less manifest. Biallelic pathogenic mutations in the *AHCY* gene have been found in all patients with SAHH deficiency.

*Statement #21. Grade of recommendation C*

In all cases with clinical suspicion of SAHH deficiency, plasma AdoMet and AdoHcy should be measured because their marked elevations in combination with normal or near normal tHcy are characteristic for the diagnosis. Plasma methionine may be normal in early infancy, but consequently likely to be constantly elevated in untreated patients. Diagnosis must be confirmed by either enzyme assay (possible in red blood cells, fibroblasts and liver) or by mutation analysis.

#### Differential diagnosis

Based on the multiorgan clinical presentation, SAHH deficiency has a very wide differential diagnosis. Once hypermethioninemia is found, further testing depends primarily on the presence of liver disease, hyperhomocysteinemia and other amino acid abnormalities. A similar constellation of metabolic findings (elevated plasma methionine, AdoMet and AdoHcy) has been reported only in ADK-deficient patients. However, in contrast to patients with SAHH deficiency, these patients have a much milder elevation of AdoMet and especially AdoHcy, with AdoMet levels being higher than AdoHcy. In addition, CK activity is increased only in some ADK-deficient patients, while in SAHH-deficient patients increased CK activity is a constant finding with much higher activity than in ADK deficiency (in SAHH deficiency almost always more than fivefold and frequently much more). Clinical presentation is also rather different in SAHH deficiency and ADK deficiency (see related sections in this article). For a related diagnostic algorithm see Fig. 2.

#### Newborn screening

Since SAHH deficiency seems to be partly treatable at least in some cases and there are indications that early diagnosis improves the outcome (Barić et al 2005; Strauss et al 2015), early identification through newborn screening could be beneficial. Currently, the only feasible way is screening for hypermethioninemia, usually done to identify CBS deficiency. Unfortunately, hypermethioninemia was present only in two out of six patients with SAHH deficiency in whom data on plasma methionine or dried blood spot methionine at the time of newborn screening was available. In two of those six patients newborn screening was performed. One of these patients had on day 3 a blood methionine level of 200 μmol/L, using the Guthrie bacterial inhibition assay (Buist et al 2006), while the other had a normal methionine level (20 μmol/L), measured by tandem mass spectrometry (Strauss et al 2015). In the remaining four patients in whom data on methionine at the time of newborn screening was available, plasma methionine was measured either because of SAHH deficiency in family members, and was normal in three patients (Barić et al 2005; I. Barić- personal communication), or because of clinical indication. In the latter case it was elevated (273 μmol/L) on day 4 (Grubbs et al 2010). A related important issue is the necessity for further diagnostic work-up following positive screening. Since increased CK activity seems to be a constant finding in SAHH deficiency, measurement of CK in dried blood spots could be an option for newborn screening, as has been used for newborn screening for Duchenne muscular dystrophy (Zellweger and Antonik 1975; Orfanos and Naylor 1984).

*Statement #22. Grade of recommendation D*

Since early treatment has shown a positive effect in some cases early identification through newborn screening may be beneficial.

*Statement #23. Grade of recommendation C*

At present, there is no sensitive screening test available to allow for newborn screening. Therefore, newborn screening is presently not possible.

#### Prenatal diagnosis

The only established way is the *AHCY* gene analysis. This is possible if the disease causing mutations are known and the carrier status of the parents has been confirmed by mutation analysis. Enzyme assay in amniocytes or chorionic villi, as well as measurement of metabolites in amniotic fluid have not been tested. The necessity for prenatal diagnosis requires thoughtful discussion, one of the reasons being the clinical variability of SAHH deficiency.

*Statement #24. Grade of recommendation D*

If the disease causing mutations are known, prenatal diagnosis is possible by *AHCY* gene analysis.

### Therapy

It seems that there are two main obstacles for the successful aetiological therapy of SAHH deficiency. The first is its multisystemic character and the second is the antenatal onset of the disease in almost all patients, with possibly irreversible consequences. Taking into account that normal biochemical findings (at least CK) have never been reported even in asymptomatic patients or in patients with late clinical onset, it is possible that biochemical abnormalities are present in all patients already *in utero*.

A *low methionine diet* can decrease and sometimes even normalize plasma AdoMet and AdoHcy, which may have positive effects on methylation and clinical and biochemical abnormalities (Barić et al 2005; Honzík et al 2012; Strauss et al 2015). It is probably justified to keep plasma methionine close to or slightly below the lower normal limit (∼10 μmol/L) to ensure the maximal possible decrease of plasma AdoMet and AdoHcy. For this purpose, patients have to be on a protein restricted diet and supplemented with a methionine-free amino acid mixture. Subsequent close clinical and biochemical monitoring seem mandatory. Phosphatidylcholine supplementation has been proposed because of expected inhibition of phosphatidylethanolamine methyltransferase by highly elevated AdoHcy, which inhibits numerous methyltransferases (Clarke and Banfield 2001). This enzyme is required for phosphatidylcholine and choline synthesis, which is important for muscles, liver and myelin. Low plasma free choline and phosphatidylcholine before treatment in the first reported patient with SAHH deficiency are consistent with the inhibition of phosphatidylethanolamine methyltransferase by the 150-fold elevation of plasma AdoHcy in that patient (Barić et al 2004), and by a study showing that a 100-fold elevation of AdoHcy decreases the activity of this methyltransferase by about 90 % (Ghosh et al 1988).

Creatine supplementation has been derived from the assumed inhibition of guanidinoacetate methyltransferase by high AdoHcy and findings of elevated guanidinoacetate in plasma or urine in some SAHH-deficient patients (Barić et al 2004; Buist et al 2006). Both phosphatidylcholine and creatine supplementation have been used in some patients, although there is no evidence that this treatment, which has been applied only in parallel with other therapeutic measures (low protein/methionine diet), results in clinical improvement. Due to the possible risk of glutathione depletion cysteine supplementation may be useful, but there is no evidence that this is really needed.

Recently, liver transplantation was successfully performed in one girl at the age of 40 months (Strauss et al 2015). The indication was insufficient success of a low methionine diet of about 35 mg/kg/day (∼2 g protein/kg/day) together with creatine and choline supplementation with aims to positively influence developmental delay and correct transmethylation homeostasis. Open to speculation was whether a more restrictive diet would have resulted in a better treatment outcome thus alleviating the need for liver transplantation. During the 6-month follow-up period, however, liver transplantation resulted in improvement of the AdoMet/AdoHcy ratio, normalization of plasma methionine and AdoMet and acceleration of head growth with promising gains in gross motor, language and social skills. The recent report on hepatocellular carcinoma in an adult with SAHH-deficiency (Stender et al 2015) provides an additional argument in favour of liver transplantation in SAHH deficiency. Longer follow-up is needed for better assessment of indications for and outcome of liver transplantation in SAHH deficiency.

The treatment outcome probably depends a lot on the severity of the disease. It is questionable whether even early intervention could help in severe cases. In others, it is possible that earlier initiation of treatment may improve the outcome. This may be the case both for liver transplantation and a low-methionine diet combined with supplementation of phosphatidylcholine and creatine. It seems that neurological, liver and coagulation abnormalities can be influenced by treatment, while the muscle disease seems as yet far less responsive or even unresponsive.

*Statement #25. Grade of recommendation C*

A *low methionine diet* is recommended since it can decrease and sometimes even normalize plasma AdoMet and AdoHcy, which in turn can have a positive effect on methylation and clinical and biochemical abnormalities. Methionine-free amino acid mixture supplementation may be necessary. Theoretically, phosphatidylcholine, creatine and cysteine supplementation seem potentially beneficial, but there is no evidence that this treatment results in clinical improvement.

*Statement #26. Grade of recommendation C*

Liver transplantation has resulted in clinical improvement as well as improvement of the AdoMet/AdoHcy ratio and normalization of plasma methionine, AdoMet and indices of increased transmethylation in a single case. Longer follow-up is needed for better assessment of the utility and indications for liver transplantation in SAHH deficiency.

### Follow-up

#### Monitoring

**Clinically**, regular careful evaluation of all body systems is indicated, particularly of the nervous system, psychomotor development, muscles, liver and coagulation. This includes imaging studies, particularly regular liver imaging. **Biochemically**, assessment of protein status, amino acids, AdoMet, AdoHcy, liver function tests, alpha-fetoprotein, CK and coagulation tests are needed. Follow-up intervals in infancy could be every 1–3 months, or even more frequently if indicated, and later every 3 to 6 months depending on the clinical course. For *patients with liver transplants* specific transplantation related follow-up regimens need to be followed.

*Statement #27. Grade of recommendation C-D*

Careful clinical and biochemical monitoring is mandatory to control both disease development and treatment to avoid their complications. The following tests are indicated: protein status, amino acids, AdoMet, AdoHcy, liver function tests, CK, alpha-fetoprotein, coagulation tests, liver imaging, while others depend on the clinical situation. Follow-up intervals depend on age and clinical course.

#### Complications

Besides the usual clinical course, complications due to bleeding diathesis, thromboembolic incidents and adaptation problems after birth due to hypotonia and respiratory insufficiency (including feeding) can be expected. An episode of liver failure has been reported once, but it is hardly possible to assess whether this was a direct consequence of the disease or due to another undefined factor. Based on occurrence in one patient with a proven diagnosis and in her sib with probable SAHH deficiency, hepatocellular carcinoma is a possible complication. Behaviour-related complications are probably to be expected more frequently than in the general population.

## Adenosine kinase deficiency

### Diagnosis

#### Selective screening

The **clinical presentation** of adenosine kinase deficiency (ADK deficiency) is multisystemic including intellectual and motor disability, muscular hypotonia, morphological abnormalities (especially frontal bossing), hepatopathy, epilepsy and recurrent hypoglycaemia (often due to hyperinsulinism). Initial symptoms are usually already evident within the neonatal period with severe or prolonged hyperbilirubinemia, hypoglycaemia and muscular hypotonia (Bjursell et al 2011; Labrune et al 1990; Staufner et al 2016a). Other signs and symptoms reported were: cardiac defects, failure to thrive and short stature, hypertelorism, strabismus, sparse hair, long and triagonal face, slender hands and feet, distorted teeth, megaloblastic anemia, retinal dystrophy and cholelithiasis.

ADK deficiency was described in 2011 (Bjursell et al 2011) and to date, 20 patients have been published in the literature (Bjursell et al 2011; Staufner et al 2016a; Labrune et al 1990—initial diagnosis was suspected to be SAHH deficiency and the correct diagnosis has been established recently). All known patients have intellectual disability ranging from moderate to very severe, frontal bossing and muscular hypotonia. Most patients develop seizures (70 %) with a median age of onset at 2 years and many have intractable epilepsy. Hepatopathy typically manifests during the neonatal period, is most prominent during the first year of life and ameliorates in the further course. It can be accompanied by severe coagulopathy mimicking acute liver failure. There is chronic liver disease with steatosis and 75 % were found to have liver fibrosis. Liver involvement is a very typical finding in ADK deficiency, but liver disease is not mandatory (Staufner et al 2016a). One patient has been diagnosed with a malignant hepatic tumour at the age of 14 years, which has an atypical histopathological appearance but most likely represents a hepatocellular carcinoma (C. Staufner—personnal communication).

Brain MRI in ADK deficiency shows delayed but ultimately complete brain maturation, which includes temporarily delayed gyral patterning and myelination. There are mostly unspecific white matter changes and potentially transient central tegmental tract hyperintensity (Staufner et al 2016b).

**Biochemical abnormalities** depend on current liver impairment. There can be elevated aminotransferases (ALAT, ASAT), direct hyperbilirubinemia and coagulation abnormalities (prolonged prothrombin time). *Specific abnormalities* are elevated plasma methionine (up to 1100 μmol/L, however methionine concentrations fluctuate and can be normal!), elevations of plasma AdoMet and AdoHcy with normal or near normal tHcy. In the reported patients, plasma AdoMet was ∼2-20-fold elevated (up to 2299 nmol/L), while AdoHcy was ∼5-30-fold elevated (up to 438 nmol/L). Adenosine in dried blood spots and urine is typically elevated, but can be normal. Two patients had mild to moderate hyperammonemia in early infancy (Staufner et al 2016a).

Clinical suspicion should be raised by the combination of some or all of the clinical signs and symptoms of the disease described above. To *confirm the diagnosis*, genetic analysis of *ADK* is recommended. To our knowledge, enzyme assays are not available.

*Statement #28. Grade of recommendation C*

In the case of clinical suspicion of ADK deficiency amino acids, AdoMet, AdoHcy and tHcy in plasma, and adenosine in urine and/or dried blood spots should be assessed. Normal concentrations of methionine and/or adenosine are frequently found and do not rule out the diagnosis. To *confirm the diagnosis* genetic analysis of *ADK* is essential.

#### Differential diagnosis

ADK deficiency may mimic the biochemical pattern of SAHH deficiency (high methionine, AdoMet and AdoHcy). However, clinical features can guide the differentiation, for example dysmorphism, neonatal hyperbilirubinemia and epilepsy are frequent in ADK deficiency but not present in SAHH deficiency. CK, AdoMet and especially AdoHcy are generally much higher in SAHH deficiency compared to ADK deficiency. Increased adenosine levels point to ADK deficiency, but are not present in all cases.

Other inborn errors of metabolism with transient neonatal cholestasis or hepatopathy are, e.g. mannose phosphate isomerase-CDG (MPI-CDG, formerly called CDG Ib), citrin deficiency, Niemann Pick disease type C, mitochondrial disorders, peroxisomal disorders, neuronopathic Gaucher disease and biliary acid synthesis disorders.

#### Newborn screening

So far, there is no sensitive marker that would allow newborn screening for ADK deficiency. Hypermethioninemia was not a consistent finding in newborn screening dried blood spots of affected patients but elevated adenosine may be an option (Staufner et al 2016a). Due to limited knowledge of the natural history and therapeutic options, newborn screening is not indicated as yet.

*Statement #29. Grade of recommendation D*

At present, newborn screening is not justified due to limited knowledge of the natural history and therapeutic options.

#### Prenatal diagnosis

Theoretically, genetic testing can be performed prenatally and the presence of known mutations of the *ADK* gene can be confirmed or ruled out. As yet, there is no experience of prenatal diagnosis in ADK deficiency.

### Therapy

There is evidence for a positive effect of a low methionine diet (reduction of the daily intake of methionine to 15–20 mg per kg of body weight) in seven patients with ADK deficiency from different centres (Labrune et al 1990; Staufner et al 2016a). Under dietary treatment the liver phenotype ameliorates clinically and biochemically, whereas a positive effect on the neurological outcome has only been reported in a single case. Decreased intake of methionine lowered the concentrations of methionine, tHcy, AdoMet and AdoHcy in plasma and adenosine in whole blood.

Diazoxide appears to be an effective treatment of recurrent hypoglycaemia caused by hyperinsulinism in ADK deficiency (Staufner et al 2016a).

Further therapeutic options yet to be explored are alternative degradation of adenosine via adenosine deaminase (ADA), which is currently available as enzyme replacement therapy for ADA-SCID patients, or caffeine treatment which can antagonize central adenosine receptors.

*Statement #30. Grade of recommendation C*

A low methionine diet should be considered as a therapeutic option. Diazoxide is recommended for recurrent hypoglycaemia when it is due to hyperinsulinism.

### Follow-up

#### Monitoring

Careful **clinical evaluation** with regular follow-up visits depending on age and severity is recommended (intervals ranging from 1 month to 1 year), including regular monitoring of psychomotor development and neurological examination and regular liver imaging. Since epilepsy is often present in ADK deficiency, regular electroencephalography is recommended. In one patient retinal dystrophy was diagnosed, thus ophthalmological examination on a regular basis should be considered. Because of an increased incidence of cardiac defects, echocardiography should be performed in all patients and followed up accordingly. Several patients presented with cholelithiasis, thus abdominal ultrasound should be performed in cases of unexplained pain (colic).

**Biochemically**, assays of protein status, plasma amino acids, tHcy, AdoMet, AdoHcy, adenosine in urine and/or dried blood spot, serum aminotransferases, total and direct bilirubin, ammonia, blood glucose, uric acid, coagulation tests and alfa-feto protein are relevant. Regular blood glucose profiles should be performed, depending on the presence and treatment of recurrent hypoglycaemia. A complete blood count should be included in the regular monitoring to check for megaloblastic anemia.

*Statement #31. Grade of recommendation C-D*

Close clinical and biochemical monitoring is needed, including monitoring of psychomotor development, liver function and hypoglycaemia.

#### Complications

Severe neonatal hyperbilirubinemia could lead to kernicterus if not treated early and aggressively. Hepatopathy can progress to liver failure and there was one case of hepatorenal syndrome. Development of a malignant hepatic tumour was observed in one patient. Recurrent hypoglycaemia, especially during the neonatal period, may be severe and can lead to further brain damage. Epilepsy can become very severe and difficult to manage. Some patients developed severe behavioural problems.

## Discussion

Although there are many specific aspects related to each of the four methylation disorders discussed in this paper, they also have some common features and some common recommendations are valid for them.

Regarding recognition and diagnostic work-up, it has to be taken into account that “hypermethioninemia”, which is the biochemical hallmark of this group of diseases, is not always present in early infancy. Accordingly, newborn screening (in countries which screen for CBS deficiency) will very likely miss a large proportion of patients and a high level of awareness of these disorders is needed to recognize affected patients in this age group. Accordingly, specific tests (AdoMet, AdoHcy, tHcy) may be indicated in all sick young infants with unexplained signs and symptoms listed above. In summary, this could be any suspicious finding or combination of unexplained neurological signs, myopathy, liver disease, dysmorphism as described in the section for ADK deficiency, unexplained elevation of aminotransferases or CK activity.

In evaluation of liver disease it should be kept in mind that hypermethioninemia is not always an unspecific secondary finding. Regardless of the current clinical presentation, every case of hypermethioninemia requires appropriate investigation (see Fig. 2), which should not be postponed since early diagnosis can improve the outcome in those patients who benefit from appropriate treatment. Unfortunately, the investigation of the key metabolites AdoMet and AdoHcy is currently performed in very few specialized metabolic laboratories. AdoMet and AdoHcy can be measured in plasma. AdoMet is known to be unstable at normal pH but rather stable under acidic conditions: the recommendation is to separate plasma as soon as possible (1 ml of plasma is sufficient). Next add to 10 parts of plasma 1 part of 1 N acetic acid and store at −20 °C (Gellekink et al 2005).^2^ Because concentrations of AdoMet and AdoHcy are in the nmol range, sensitive LC-MS/MS methods have to be employed. Whole blood analyses can be inaccurate. Homocysteine should be measured in plasma as tHcy, which is available in most clinical chemistry laboratories. The assays for amino acids analyses usually do not detect moderately elevated tHcy.

Considering the complex pathogenesis involving an uncertain number of essential methylation processes, treatment options for methylation disorders, with the exception of MAT I/III deficiency, can hardly be comprehensive. One common aspect is the lack of evidence that infections, surgery or other short catabolic periods may cause significant problems to the patients in comparison to healthy children. Accordingly, there is no evidence in support of a need to avoid catabolic periods or for specific emergency treatment. Due to the complexity of treatment and follow-up it is recommended that all patients with methylation disorders are treated and monitored under the surveillance of specialized centres. The limited number of patients renders collaborative studies difficult, but important.

Finally, it should be taken into account that the recommendations provided in this publication are based on general principles and only a very limited number of patients and publications, and in practice should be adjusted individually according to patient’s age, severity of the disease, and clinical and laboratory findings. Much more experience is necessary to offer more accurate answers to many remaining questions and uncertainties of methylation disorders including long-term disease manifestations and complications. One important tool for gaining these experiences are registries like the “E-HOD (European network and registry for homocystinurias and methylation defects)” registry (see https://www.ehod-registry.org/ and http://www.e-hod.org/). We would like to encourage all care-takers to include their patients with methylation disorders in the “E-HOD” registry, which would enable the development of better guidelines of methylation disorders in the future.

*Statement #32. Grade of recommendation C-D*

According to current knowledge, there is no need for a specific regimen in catabolic situations for any of the four methylation disorders.

## Electronic supplementary material

Below is the link to the electronic supplementary material.

ESM 1 (DOCX 13 kb)

## Abbreviations

ADK: Adenosine kinase
AdoHcy: S-adenosylhomocysteine
AdoMet: S-adenosylmethionine
CBS: Cystathionine beta-synthase
CK: Creatine kinase
GNMT: Glycine N-methyltransferase
MAT: Methionine adenosyltransferase
MRI: Magnetic resonance imaging
SAHH: S-adenosylhomocysteine hydrolase
tHcy: Total homocysteine

## Footnotes

1 MAT I/III deficiency is also referred to as Mudd’s disease in recognition of the late Harvey Mudd’s contribution to the knowledge of hypermethioninemias in general and specifically to his efforts in producing the key review manuscript (Chien et al 2015) only a couple of weeks before he passed away.

2 Laboratories where measurements of AdoMet and AdoHcy are available: Laboratory of Clinical Biochemistry and Metabolism, Department of General Pediatrics Adolescent Medicine and Neonatology, University Medical Centre Freiburg, Freiburg, Germany, contact person- Henk Blom, e-mail: henk.blom@uniklinik-freiburg.de; Institute of Inherited Metabolic Disorders, Charles University in Prague-1st Faculty of Medicine, Prague Czech Republic, contact person- Viktor Kozich, e-mail: viktor.kozich@lf1.cuni.cz; University of Colorado, Department of Medicine/Division of Hematology, Aurora, Colorado, contact person Sally P. Stabler, e-mail: Sally.Stabler@ucdenver.edu
