# Supplementary material for: Consensus recommendations for the diagnosis, treatment and follow-up of inherited methylation disorders
Source: J Inherit Metab Dis. 2016 Sep 26;40(1):5–20. doi: 10.1007/s10545-016-9972-7 (PMC5203850; doi:10.1007/s10545-016-9972-7)
Supplement: Supplementary file 1 — (DOCX 13 kb) [file 10545_2016_9972_MOESM1_ESM.docx]

**Supplementary material**

Literature evaluation according to SIGN methodology

According to SIGN (Scottish Intercollegiate Guideline Network, <http://www.sign.ac.uk>) methodology all evaluated articles on MAT I/III, GNMT, SAHH and ADK deficiency had level of evidence 3 or 4. This was the basis for consensus conclusions and recommendations which have been graded in the following way:

A If level 1 evidence was found (not the case).

B If level 2 evidence was found.

C If level 3 evidence was found (mainly non-analytical studies such as case reports and

case series).

D If level 4 evidence was found (mainly expert opinion).

The process of generating recommendations in this paper

The process of generating these recommendations started with the kick-off meeting in 2013, followed by the literature search and evaluation in the next year, drafting of the manuscript, and co-authors draft evaluation meeting in September 2015. After that the manuscript was revised and internal final agreement reached. After external reviewers (Martina Huemer, Division of Metabolism and Children's Research Center, University Childrens' Hospital Zürich, Zürich, Switzerland & Department of Paediatrics, Landeskrankenhaus Bregenz, Bregenz, Austria; Brian Fowler, Division of Metabolism and Children's Research Center, University Childrens' Hospital Zürich, Zürich, Switzerland & University Childrens' Hospital Basel (UKBB), Basel, Switzerland) comments were collected, the final revision has been produced and submitted for publication.
